# ACE-inhibition induces a cardioprotective transcriptional response in the metabolic syndrome heart

**DOI:** 10.1038/s41598-018-34547-9

**Published:** 2018-11-01

**Authors:** Aziza Yakubova, Lieven Thorrez, Dmitry Svetlichnyy, Liesbeth Zwarts, Veerle Vulsteke, Griet Laenen, Wouter Oosterlinck, Yves Moreau, Luc Dehaspe, Jeroen Van Houdt, Álvaro Cortés-Calabuig, Bart De Moor, Patrick Callaerts, Paul Herijgers

**Affiliations:** 1 Department of Cardiovascular Sciences, Research Unit of Cardiac Surgery, KU Leuven, Leuven, Belgium; 2 Department of Development and Regeneration, Interdisciplinary Research Facility, KU Leuven, Campus Kulak Kortrijk, Kortrijk, Belgium; 3 Department of Human Genetics, Laboratory of Computational Biology, KU Leuven, Leuven, Belgium; 4 Department of Human Genetics, Laboratory of Behavioral and Developmental Genetics, KU Leuven, Leuven, Belgium; 5 Department of Electrical Engineering, ESAT - STADIUS, Stadius Centre for Dynamical Systems, Signal Processing and Data Analytics, KU Leuven, Leuven, Belgium; 6 Department of Human Genetics, Genomics Core, Center for Human Genetics, University Hospital, KU Leuven, Leuven, Belgium

## Abstract

Cardiovascular disease associated with metabolic syndrome has a high prevalence, but the mechanistic basis of metabolic cardiomyopathy remains poorly understood. We characterised the cardiac transcriptome in a murine metabolic syndrome (MetS) model (LDLR−/−; ob/ob, DKO) relative to the healthy, control heart (C57BL/6, WT) and the transcriptional changes induced by ACE-inhibition in those hearts. RNA-Seq, differential gene expression and transcription factor analysis identified 288 genes differentially expressed between DKO and WT hearts implicating 72 pathways. Hallmarks of metabolic cardiomyopathy were increased activity in integrin-linked kinase signalling, Rho signalling, dendritic cell maturation, production of nitric oxide and reactive oxygen species in macrophages, atherosclerosis, LXR-RXR signalling, cardiac hypertrophy, and acute phase response pathways. ACE-inhibition had a limited effect on gene expression in WT (55 genes, 23 pathways), and a prominent effect in DKO hearts (1143 genes, 104 pathways). In DKO hearts, ACE-I appears to counteract some of the MetS-specific pathways, while also activating cardioprotective mechanisms. We conclude that MetS and control murine hearts have unique transcriptional profiles and exhibit a partially specific transcriptional response to ACE-inhibition.

## Introduction

Cardiovascular disease prevalence and associated mortality is higher in individuals with metabolic syndrome (MetS)^1,2^. The International Diabetes Federation (IDF) defines MetS as central obesity plus any two of the following: elevated plasma triglyceride (TG) levels (≥150 mg/dl), reduced high-density lipoproteins (HDL) (<40 mg/dl for men and <50 mg/dl for women), increased blood pressure (≥130 mmHg systolic or ≥85 mmHg diastolic), and/or increased fasting plasma glucose (≥100 mg/dl)^3^. According to these criteria, MetS affects 20–25% of the world’s population. Individuals with MetS have a twofold higher risk of having coronary heart disease (CHD) and myocardial infarction due to development of metabolic cardiomyopathy^4^.

Only a few studies defined the phenotypic characteristics of metabolic cardiomyopathy^5,6^. Previous research in our group characterized a double knock-out mouse model (LDLR−/−; ob/ob, called DKO henceforth)^7^ that mimics many of the characteristics of patients with metabolic syndrome. The mouse model exhibits impaired left ventricular contraction and increased ventricular stiffness with increased myocardial fibrosis. Preload recruitable stroke work, a load independent parameter of contractility, was 35% lower in DKO than in wild type mice (WT). End diastolic pressure volume relationship (EDPVR) was 50% higher in DKO than in WT. Arterial elastance (Ea), a parameter of ventricular afterload was 42% higher in DKO than in WT. The metabolic profile shows severe insulin resistance (with HOMA-IR 10-fold higher in DKO), a more than double glycemia level, a more than sevenfold total cholesterol level, and more than tenfold level of blood triglycerides. Contractile reserve was significantly reduced in DKO. Body weight at 24 weeks was more than double in DKO, but heart weight corrected for tibia length only increased by 7%. At the cellular level, we reported that cardiomyocytes in the MetS heart demonstrate slower kinetics of contraction and relaxation^8^.

Despite the extensive pathophysiological characterization of the MetS heart, the molecular mechanisms governing metabolic cardiomyopathy remain poorly understood. The apparent obstacle is the complex, interdependent multifactorial nature of the metabolic syndrome itself. The need for a better understanding of molecular disease mechanisms is further substantiated by the limitations of conventional therapies aimed at single therapeutic targets.

The European Society of Cardiology recommended the use of genomic methods and bioinformatics tools to investigate the full transcriptomic heart profile in order to characterize the signalling networks accountable for cardioprotection, and to identify potential therapeutic targets for use in multitarget treatment strategies^9^. The use of genomic diagnostic tools can also lead to early identification of individuals at increased risk and to novel therapeutic strategies that can reduce acute ischemic damage and remodelling towards heart failure in advanced disease.

The heart is composed of different cell types, where cardiac myocytes account for only about a third of the total cell number^10^. The rest includes a broad range of other cell types, including fibroblasts and other connective tissue cells, smooth muscle and endothelial cells and immune system–related cells^11–13^. These distinct cell groups are not isolated from one another within the heart, but interact via paracrine, autocrine and endocrine factors. Therefore, the impact of cellular crosstalk should be considered to understand pathogenic disease mechanisms and therapeutic effects. In our study, the use of genomic tools in examining whole heart samples contributes to understanding heterocellular cell-cell interactions in metabolic cardiomyopathy and treatment.

Angiotensin-converting enzyme inhibition (ACE-I) is a therapy of choice for patients with MetS^14,15^, along with other pharmacologic agents^16^. It has been shown that ACE-I ameliorates some of the MetS pathogenic characteristics, as it exerts an anti-hypertensive effect and improves glucose control and insulin sensitivity^17,18^. We reported earlier that ACE-I by captopril also improves cardiac contractility in the MetS murine model^8,19^. Glycemia in DKO treated with ACE-I was 23% lower than in untreated controls, total cholesterol was 14% lower, and triglycerides were 21% lower. ACE-I had no effect on body weight. Heart weight corrected for tibial length in DKO was reduced by 13%. Left ventricular load independent contractility determined by preload recruitable stroke work was unchanged in WT, but improved by 16% in DKO, while EDPVR was unchanged in WT, but normalized in DKO (50% reduction). Arterial elastance reduced 31% in WT and 50% in DKO, proving the effectiveness of the applied dose of captopril. Contractility and contractile reserve of isolated cardiomyocytes from DKO mice was partly restored after ACE-I. However, our understanding of the effects of ACE-I treatment on the heart and metabolic cardiomyopathy remains very limited.

The goal of the current study was to characterize the transcriptional signature in the heart of a murine model of MetS relative to a healthy, control heart and to characterize the transcriptional changes induced by ACE-I in control and MetS hearts.

## Results

### Transcriptional characterization of the heart of a murine MetS model

The DKO mouse is a clinically representative model for MetS, which enables the study of cardiovascular consequences and therapeutic interventions and was extensively phenotyped in previous studies^7,8,20^. The phenotypes in DKO mice are highly consistent across individuals and include higher body weight, glucose, insulin, total cholesterol levels, plasma triglycerides, pulse pressure, and mean arterial blood pressure, elevated insulin resistance and impaired glucose tolerance. DKO mice develop metabolic cardiomyopathy from the age of 24 weeks. The cardiomyopathy is characterized by impaired contractility and relaxation, increased end-diastolic ventricular stiffness, increased ventricular myocardial fibrosis, and decreased cardiac output. DKO hearts show higher collagen content, increased cardiac myocyte cell width, and the presence of atherosclerotic lesions.

**Figure 1:**
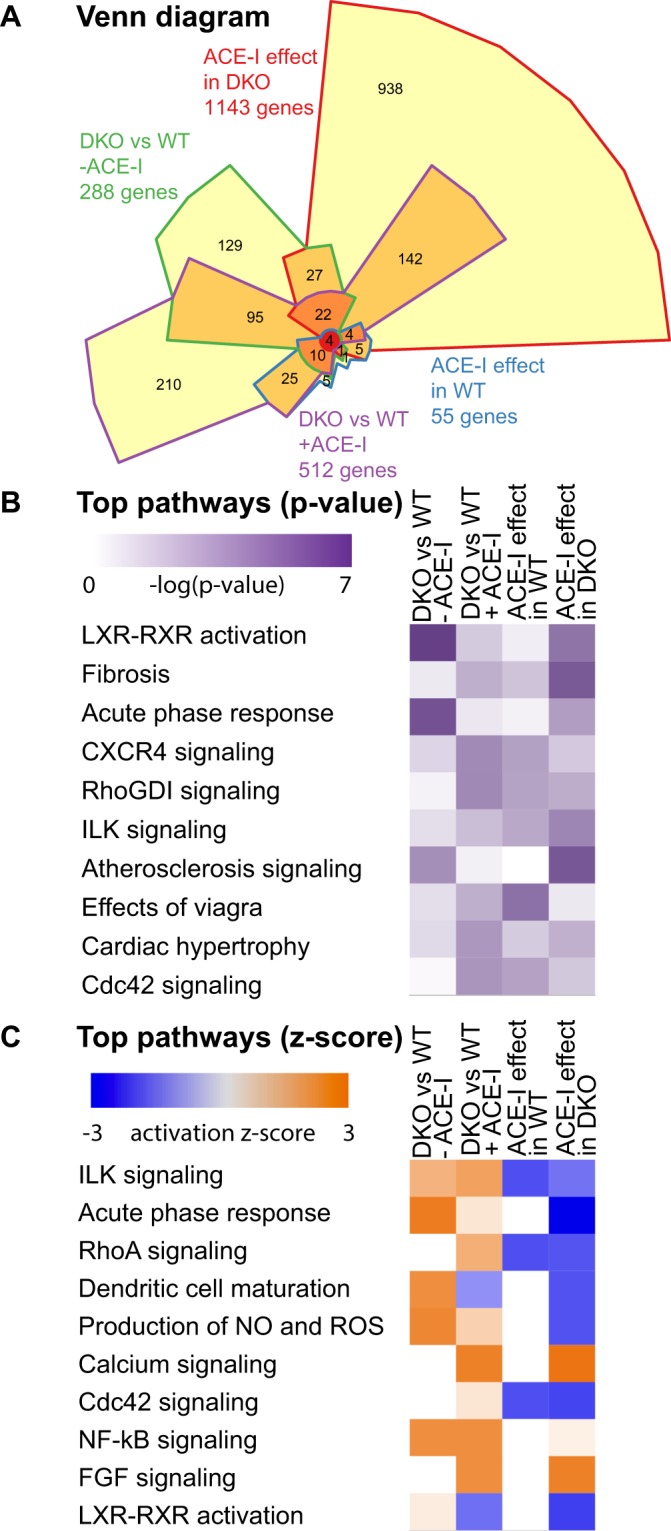
(**A**) Chow-Ruskey Venn diagram of number and overlap of differentially expressed genes when comparing WT to DKO mice and when comparing the effect of ACE-I in both animal models. (**B**) Comparison analysis of top 10 pathways most affected based on p-value (relative number of genes differential versus total members of the pathway). (**C**) Comparison analysis of top 10 pathways most severely up (orange) or down (blue) regulated. Observed up or down regulation is compared to known changes that are either activating or inhibiting, as derived from the literature and compiled in the Ingenuity® Knowledge Base.

RNA-seq analysis of 39,175 genes identified 288 genes that are differentially expressed between DKO-CO and WT-CO hearts (Fig. 1A). We used Ingenuity Pathway Analysis (IPA) to integrate and help interpret our expression data and found 72 significantly enriched pathways (top 10 shown in Fig. 1B,C, the complete list in Suppl. Table S1), with the majority of pathways indicating an increase in activity. We have opted to combine in the figures the comparison DKO versus WT (panels A where appropriate) with the comparison DKO versus DKO-ACE-I (panels B where appropriate). This was done to allow easy comparison of (i) how pathways are disturbed in DKO and (ii) reverted or altered upon treatment with ACE-I.

**Figure 2:**
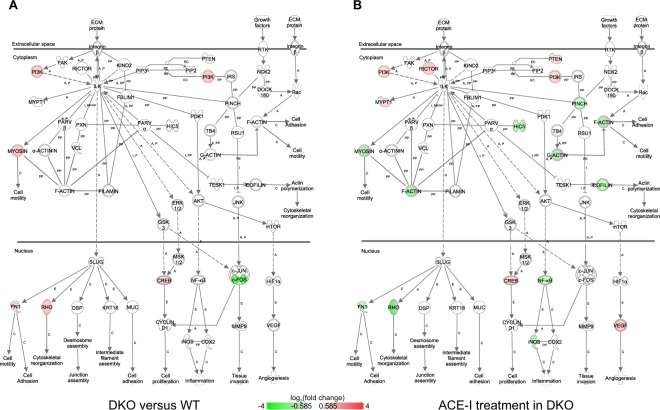
Integrin linked kinase (ILK) signaling. This pathway illustrates cytoskeletal changes and effects through integrin contacts with the extracellular matrix. (**A**) Differential gene expression in DKO mice versus WT mice. Red = upregulated, green = downregulated. A = activation, C = causes, E = Expression, EC = Enzyme Catalysis, I = inhibition, PP = Protein-Protein binding, RE = Reaction. (**B**) Differential gene expression in DKO mice after ACE-I. Red = upregulated, green = downregulated. A = activation, C = causes, E = Expression, EC = Enzyme Catalysis, I = inhibition, PP = Protein-Protein binding, RE = Reaction.

**Figure 3:**
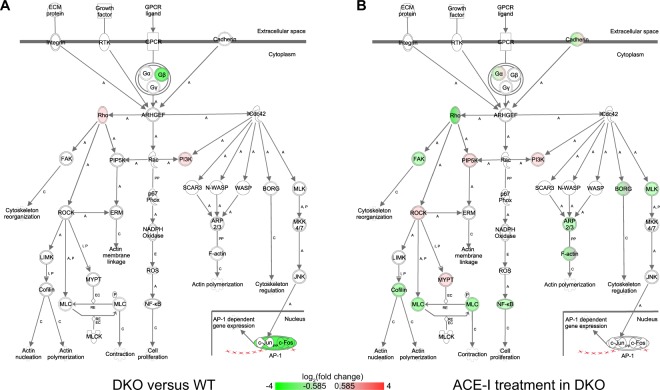
Rho GTPase family signaling. (**A**) Differential gene expression in DKO mice versus WT mice. (**B**) Differential gene expression in DKO mice after ACE-I. GTPases regulate a wide range of biological processes. The mammalian GTPase family currently consists of three subfamilies: Rho, Rac and Cdc42 and each affect numerous downstream molecules that mediate effects on the cytoskeleton and gene expression. The small GTPases act as molecular switches, cycling between an active GTP-bound state and an inactive GDP-bound state. A number of proteins have been identified as targets of Rho with ROCK being a prominent target. ROCK phosphorylates MLC, which plays an important role in actomyosin contractility. ROCK also activates LIMK, which results in Cofilin inactivation and leads to actin polymerization. Rac and Cdc42 also bind to PI3K, thus activating the PI3K/AKT signaling pathway. Red = upregulated, green = downregulated. A = activation, C = causes, E = Expression, EC = Enzyme Catalysis, I = inhibition, P = (de−)Phosphorylation, PP = Protein-Protein binding, RE = Reaction.

First, our findings implicated activation of pathways not previously associated with the MetS heart. These include ILK signalling, dendritic cell maturation, and production of nitric oxide and reactive oxygen species in macrophages (Fig. 1B,C). In the ILK signalling pathway (Fig. 2A) our analysis indicated upregulated expression of *Ras homolog family member* (*Rho*), *myosins*, and *fibronectin 1* (*Fn1*). Furthermore, in addition to the upregulation of *Rho*, we observed downregulated expression of the transcription factor (TF) *Fos* in the ILK and in the Rho signalling pathways (Figs 2A,3A).

**Figure 4:**
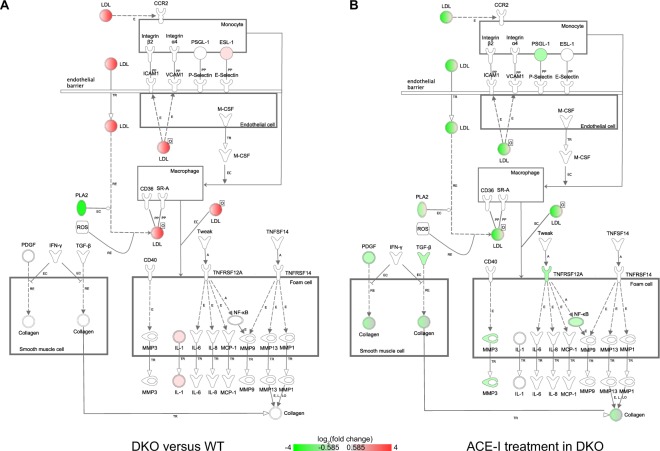
Atherosclerosis signaling. (**A**) Differential gene expression in DKO mice versus WT mice. (**B**) Differential gene expression in DKO mice after ACE-I. Monocytes adhere to endothelial cells and migrate into the intima of the blood vessel where they differentiate into macrophages. Atherogenic lipoproteins such as oxidized LDL play an initiating role in this adhesion. Macrophages take up oxidized LDL through scavenger receptors on their cell surface to form foam cells. Such lipid laden foam cells in the intima form a “fatty streak”. Foam cells secrete pro-inflammatory cytokines as well as MMPs. This in turn amplifies the local inflammatory response and stimulates SMC proliferation and migration toward the lesion, leading to formation of the thick fibrous cap of the stable plaque. Red = upregulated, green = downregulated. E = Expression (includes metabolism/synthesis for chemicals), EC = Enzyme Catalysis, L = Molecular cleavage or degradation, LO = Localization, O = Oxidized, PP = Protein-Protein binding, RE = Reaction, TR = Translocation.

**Figure 5:**
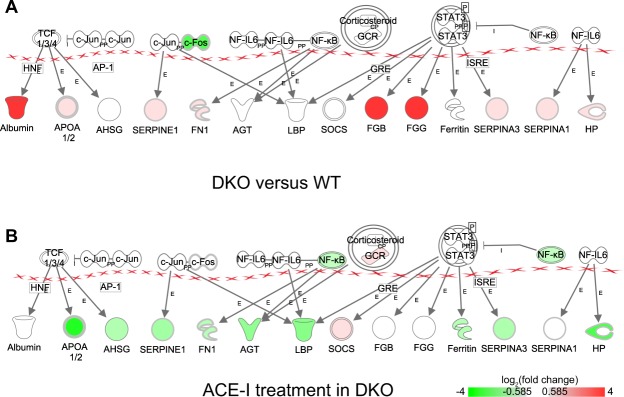
Acute phase response. (**A**) Differential gene expression in DKO mice versus WT mice. (**B**) Differential gene expression in DKO mice after ACE-I. The acute phase response is a rapid inflammatory response that can –amongst others- be triggered by tissue injury. It consists of an increase in inflammatory factors and a change in concentration of several plasma proteins (the acute phase proteins). Red = upregulated, green = downregulated. E = Expression, I = inhibition, PP = Protein-Protein binding.

Second, our findings identified differentially expressed genes that have not been described in this context before. For instance, in the cardiac hypertrophy signalling pathway (Suppl. Fig. S1A), we observed upregulated expression of *Rho*, *Adenylate cyclase* (*Ac*), *cAMP-dependent protein kinase* (*Pka*), and downregulated expression of *heat shock protein 27* (*Hsp27*). In the LXR-RXR (Suppl. Fig. S2A) and atherosclerosis (Fig. 4A) signalling pathways our results showed upregulated expression of *low-density lipoprotein* (*Ldl*), *high-density lipoprotein* (*Hdl*), *ATP binding cassette subfamily A member 1* (*Abca1*), *ATP binding cassette subfamily G member 1* (*Abcg1*), *Abcg4*, *interleukin 1 alpha* (*Il-1), E-selectin ligand-1* (*Esl-1*) and downregulated expression of *phospholipase A2* (*Pla-2*). In the acute phase response pathway (Fig. 5A), we observed downregulated expression of the TF *Fos* and upregulated expression of *albumin, serpin 1, serpin 3, apolipoprotein A-I* (*Apoa1/2*), *fibrinogen beta chain* (*Fgb*), *fibrinogen gamma chain* (*Fgg*), and *haptoglobin* (*Hp*). The differential expression of a selection of genes from these pathways was validated by means of qRT-PCR (see Suppl. Table S2).

**Figure 6:**
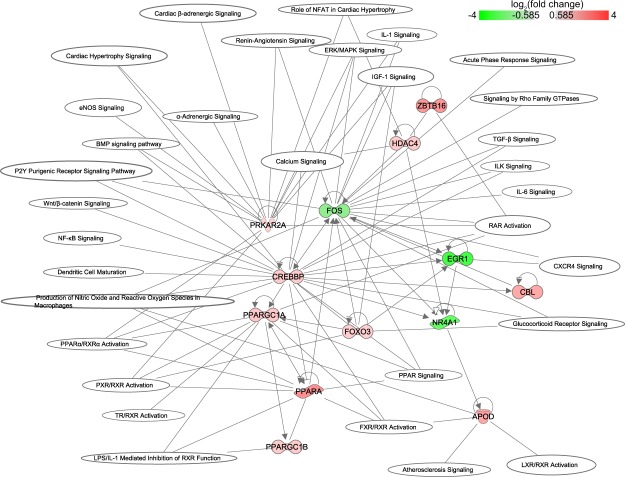
Pathways significantly changed when comparing DKO with WT mice. Only differential transcription factors are plotted in a network representation to show involvement in pathways. Red and green color indicate up- and downregulation, respectively.

**Figure 7:**
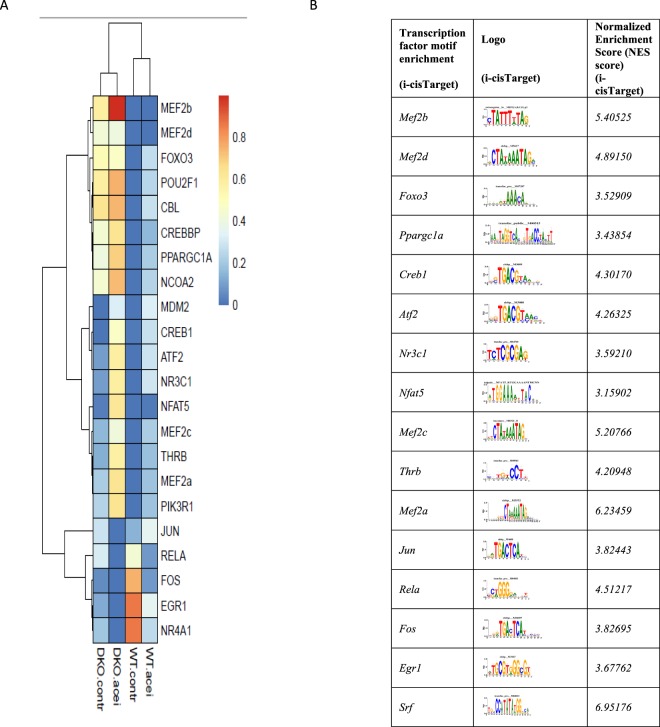
(**A**) Gene expression activity for IPA transcription factor prediction for all 4 conditions. FPKM values have been scaled across experimental conditions for convenient visualization ranging from low (0) to high (1) relative expression levels; (**B**) i-Cis-Target based transcription factor binding site enrichment analysis.

In a network representation, we displayed the differentially expressed TFs and their involvement in several pathways (Fig. 6). The TFs *Fos*, *Foxo3*, *Crebbp*, *Ppargc1, Prkar2a* and *Egr1* are involved in most of the pathways. Additional *i-cis* Target analysis confirmed that the up- and downregulated genes are enriched for binding site motifs of these transcription factors (Fig. 7). The expression of these transcription factors in control and MetS hearts was confirmed by means of RT-PCR (see Suppl. Fig. S3).

### ACE-I induces a model-specific transcriptional response in control hearts

To gain insight in the transcriptional effects of ACE-I treatment, we first characterized the impact of ACE-I on the transcriptome of control hearts. To this end, we compared the transcriptomes of whole hearts of captopril treated (WT-AI) and untreated WT mice (WT-CO).

We found that ACE-I in WT mice had a limited effect on gene expression. RNA-seq analysis of 39,175 genes revealed only 55 genes differentially expressed between WT-AI and WT-CO hearts (Fig. 1A). This is a relatively small effect, when compared to changes due to WT versus DKO genotype (288 genes). IPA analysis of these genes revealed 23 significantly enriched pathways (Fig. 1B,C) with an increase in activity level in one and a decrease in four pathways, all of which were related to Rho signalling. Importantly, we identify numerous genes that have not previously been implicated in the effect(s) of ACE-I treatment. In a network representation showing the connection between the differentially expressed genes and the enriched pathways, we observed only one TF, *Fos*, which is significantly downregulated (Suppl. Fig. S4).

Downstream targets of Fos were displayed in the network: *Nr4a1, Bmp10*, *insulin-like growth factor-binding protein 6* (*Igfbp6), natriuretic peptide* (*Nppa*), *dickkopf-related protein* 3 (*Dkk3*), all with a decrease in expression (Suppl. Fig. S4). Besides *Fos*, the network representation revealed three genes involved in most of the pathways: *myosin light chain 1, 4 and 7* (*Myl1, Myl4, Myl7*) (Suppl. Fig. S4). The expression of differentially expressed genes was validated by means of qRT-PCR (see Suppl. Table S3). Noteworthy, IPA transcription factor prediction for downregulated genes was consistent with the results we obtained via *i-cis*Target (Fig. 7). The *i-cis* Target analysis did not predict any TF binding site enrichment for up-regulated genes.

### ACE-I induces a model-specific transcriptional response in MetS hearts

To characterize transcriptional changes induced by ACE-I in MetS heart and to identify key pathways with possible roles in the observed improvement in cardiac phenotype, we compared the transcriptome between ACE-I treated and untreated DKO hearts (DKO-AI vs. DKO-CO).

In DKO hearts, ACE-I resulted in a very prominent transcriptional response compared to WT hearts. Specifically, out of 39,175 genes analyzed, 1143 genes were differentially expressed between DKO-AI and DKO-CO hearts (Fig. 1A). IPA analysis of these genes revealed 104 significantly enriched pathways with the majority of pathways displaying a decrease in overall activity (top 10 shown in Fig. 1B,C, the complete list in Suppl. Table S1).

The first important observation was that the top pathways with a decrease in activity could be subdivided in two groups. The first group consisted of pathways associated with Rho signalling, such as regulation of actin-based motility by Rho, Cdc42 signalling, RhoA signalling, signalling by Rho Family GTPases, Integrin signalling, and ILK signalling pathways.

The second group included pathways associated with adaptive and innate immune system response and inflammation, including acute phase response signalling, PKCθ signalling in T-lymphocytes, dendritic cell maturation, production of nitric oxide and reactive oxygen species in macrophages, complement system, and leukocyte extravasation signalling.

The second important observation was that ACE-I in DKO mice resulted in enrichment of pathways not enriched in untreated DKO hearts and in further modification of pathways characteristic for untreated DKO hearts. The top ACE-I newly induced pathways were associated with Rho-signalling, adaptive and innate immune system (Suppl. Fig. S5).

In addition to these pathways, ACE-I changed the activity of acute phase response signalling, LXR-RXR activation, production of nitric oxide and reactive oxygen species in macrophages, and dendritic cell maturation pathway, from increased in DKO-CO to decreased in DKO-AI after ACE-I. Consistent with this, we confirmed by qRT-PCR the downregulation of IL-1, serpin and Fgg, genes that were upregulated in DKO relative to WT (see Suppl. Tables S4 and S2).

In DKO, ACE-I affected ILK signalling, Rho signalling, and cardiac hypertrophy signalling (Figs 2B, 3B, Suppl. Fig. S1B). Importantly, whereas ACE-I resulted in a 2 to 3-fold downregulation in WT of myosin light chain subunits *Myl1*, *Myl4*, and *Myl7*, only a modest or no downregulation was observed in DKO upon ACE-I (Suppl. Tables S3 and S4 for qRT-PCR results). In the ILK signalling pathway, ACE-I resulted in upregulation of *Rictor* and downregulation of F-actin (Fig. 2B and Suppl. Table S4 for qRT-PCR results).

ACE-I treatment impacted the LXR-RXR (Suppl. Fig. S2B) and atherosclerosis (Fig. 4B) signalling pathways. Since several of the genes associated with these pathways were upregulated in the DKO heart compared to the WT heart (as confirmed by qRT-PCR for *ApoB* ( = Ldl), *IL-1*, *Fgg*, *Pla2g1b*, *Pla2g7*), the apparent effect of ACE-I in DKO is the partial reversal of gene expression changes typical for the DKO model.

In the acute phase response (Fig. 5B) pathway, ACE-I treatment resulted in a change of gene expression from upregulation in DKO-CO (versus WT-CO) to downregulation in DKO-AI (versus DKO-CO).

Differentially regulated TFs were shown in relation to all pathways affected by ACE-I in DKO (Suppl. Fig. S5). Transcription factors *Rela, Egr1, Socs, Nr3c1, Mef2a, Nfat5, Creb1, Atf2*, and *Thrb w*ere predicted to be involved in most of the pathways affected by ACE-I in DKO (Suppl. Fig. 5). The gene *Myl9* was also included in this network representation. In the network, *Myl9* is involved in many of the affected pathways including signalling by Rho family GTPases. Additional *i-cis* Target analysis confirmed binding site enrichment of these transcription factors in the set of up- and downregulated genes (Fig. 7).

## Discussion

**Figure 8:**
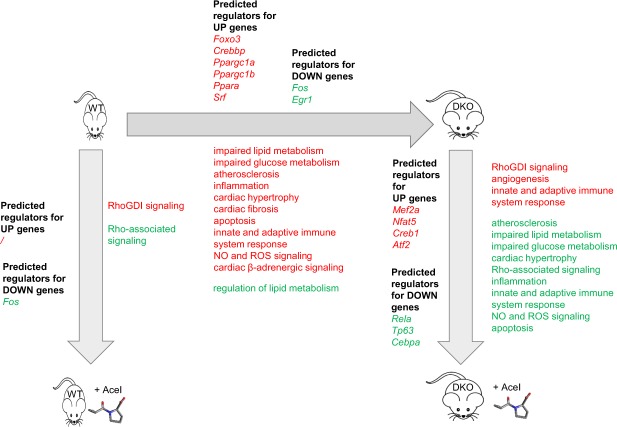
Schematic representation of main effects on pathways in the metabolic syndrome hearts (DKO) versus WT hearts and for both mouse models the effect of ACE-I. Red = upregulated pathway, green = downregulated pathway.

In our study, we characterized the differences in cardiac transcriptome between a murine model of MetS and healthy controls and determined the impact of ACE-I treatment on control and MetS hearts. Whole heart was used to capture the cellular complexity and intercellular interactions in metabolic cardiomyopathy and treatment. We identify pathways and gene networks not previously associated with MetS heart, or with ACE-I effects on healthy and MetS hearts, while also providing a detailed description of gene activity patterns and networks for pathways that had been implicated before. A schematic overview of our findings is shown in Fig. 8.

Thus far, the transcriptional landscape of MetS hearts has remained largely unknown. A few studies - using the Zucker Diabetic Fatty (ZDF) rat model - have reported differential expression of a few genes associated with impaired lipid and glucose metabolism, atherosclerosis, ROS signalling, and apoptosis^21^. Our results reveal that the MetS cardiac transcriptome is significantly different from controls for 288 genes and 72 pathways. Several of these pathways have previously been implicated directly or indirectly in MetS cardiomyopathy, while a number of the most significantly affected pathways are novel but can be linked to the morphological and functional phenotypes seen in our MetS model.

Several of the novel pathways identified here, converge on Rho signaling. Rho signaling and its downstream effector, Rho-kinase (ROCK) have been causally implicated in angiotensin-induced cardiac remodelling and hypertension^22^. Furthermore, myosin phosphatase target subunit 1 (MYPT1) has an important role in ROCK-regulated vascular smooth muscle cell (VSMC), and non-muscle cell contraction^23,24^. Specifically, phosphorylation by ROCK inactivates MLC phosphatase and allows interaction of MLC/MYL with actin^25^. We propose that the changes in Rho signalling contribute to altered contractility and other cardiac changes in MetS hearts.

We also identify innate and adaptive immune system signalling pathways. Our findings contribute to recent studies that link the innate and adaptive immune system to cardiovascular dysfunction in obesity^26,27^. Insulin resistance was reported to be associated with infiltration of macrophages into cardiovascular tissues, followed by enhanced M1 pro-inflammatory response and suppression of M2 anti-inflammatory responses thus promoting cardiac hypertrophy, inflammation, and fibrosis^28^. The phenotypic switch of macrophages in obesity is associated with cytokine production^29^. Induction of pro-inflammatory cytokines IL-1 and IL-6 by angiotensin II has been suggested to cause cardiac hypertrophy^30^. In our study, we found IL-1, but not IL-6 to be significantly upregulated in DKO versus WT. Treatment with ACE-I resulted in a significant reduction of IL-1 expression and could thus be a contributing factor to the improved cardiac performance observed following ACE-I treatment.

We also found changes in pathways related to LXR-RXR signalling, atherosclerosis, and acute phase response that have been implicated before in cardiac pathophysiology^31–35^. Our results implicate these pathways in development and progression of MetS associated cardiomyopathy.

In addition to the pathway analyses, we studied transcription factors associated with the observed transcriptional changes. We found a threefold upregulation of *Srebp1c* between DKO and WT that, however, did not reach statistical significance (FDR corrected p-value of 0.054). We observed significant upregulation of *Foxo3*, but not *Foxo1*, *Foxo4* and *Foxo6*. We found *Pparα* as well as *Ppparγ Coactivator 1 Alpha and Beta (Pppargc1a* and *Pppargc1b*) to be upregulated in DKO versus WT, while *Ppparγ* was not. SREBP1c and FOXO have been reported to play a role in transcriptional regulation of metabolism at high glucose levels, FOXO-1 in cardiac pathology in diabetes mellitus, PPARγ in lipid metabolism, and PPARs in cardiac dysfunction in diabetic cardiomypathy^36–38^. Our findings implicate these and related transcription factors in the MetS heart.

In addition to studying the cardiac transcriptional changes associated with MetS, we characterized the transcriptional impact of ACE-I treatment in control and in MetS hearts. ACE-I treatment is a therapy of choice for patients with MetS, that we showed before to also improve cardiac physiological and metabolic parameters in our mouse model^8,19^. The transcriptional changes induced by ACE-I treatment in WT hearts have never been documented, while the impact of ACE-I treatment in MetS hearts is poorly understood.

The effect of ACE-I on gene expression in WT hearts is limited. Nevertheless, the downregulated expression of myosin light chains *Myl1, Myl4*, and *Myl7* in response to ACE-I provides a molecular explanation for the improved cardiac contractility in the WT heart, previously reported by our group^8,19^. Indeed, overexpression of myosin light chain proteins has been shown to play an important role in hypertension, cardiac remodelling and hypertrophy^39–41^.

Contrary to the limited effect on gene expression in WT, ACE-I in MetS hearts resulted in a very prominent transcriptional response. The majority of significantly enriched pathways displayed an overall decrease in activity after ACE-I. Interestingly, the majority of these pathways had increased activity in DKO relative to WT. Therefore, we conclude that ACE-I treatment partially reverses the DKO signature resulting in the observed improvement in cardiac morphological and functional parameters. The molecular changes include the previously discussed Rho-signalling, and adaptive and innate immune system pathways as well as the *Myl* genes. ACE-I also counteracted changes known to be characteristic for MetS. In particular, downregulation of *Ldl (ApoB)* and *IL-1* was observed, affecting the LXR-RXR and atherosclerosis signalling pathways. We also found upregulation of *rictor*, a gene encoding a subunit of the mTORC2 complex and active in the ILK signaling pathway. This is of particular interest since mTORC2 has previously been implicated in cardioprotective mechanisms^42–44^.

At the transcription factor level, *Fos* is the key TF regulating the pathways modified by ACE-I in the WT heart. In the MetS heart, the massive transcriptional changes were coordinated by several other TFs: *Rela, Egr1, Socs, Nr3c1, Mef2a, Nfat5, Creb1, Atf2, and Thrb*. In MetS hearts, ACE-I did not decrease *Fos* expression contrary to what we observed in WT cardiac transcriptome. It seems likely that the prominent transcriptional changes associated with metabolic syndrome heart as compared to the wildtype heart on the one hand, and the changes induced upon captopril treatment on the other hand, will also involve alterations at the epigenetic level. Recent studies have highlighted the importance of epigenetic regulation in heart disease (see e.g.^45,46^). The presence in the networks of histone deacetylases (*HDAC4*, *HDAC9* in Figs 6 and S5, respectively) suggests that this will also play a role in the MetS heart model molecularly characterized in the current manuscript.

In summary, our study provides novel insights into the cardiomyopathy associated with metabolic syndrome and the effects of ACE-I treatment. First, our study characterized the transcriptional landscape in the heart of a murine model of metabolic syndrome relative to a healthy, control heart. Second, we characterized the transcriptional changes induced by ACE-I in the control and in the MetS hearts where it activates cardioprotective mechanisms. The observed changes correspond to our previous observations at the phenotype level and they provide a mechanistic explanation for those results. In conclusion, our results identify different deleterious and protective pathways not previously implicated in the cardiomyopathy of metabolic syndrome and thereby define possible novel avenues for studies in patients with MetS.

## Materials and Methods

### Experimental animal models

The study was conducted on a double knockout murine model of MetS (DKO; LDLR−/− and ob/ob)^47^. C57BL/6 (WT) mice were used as control. DKO mice were backcrossed for at least 10 generations into the C57BL/6J background, resulting in mice having a 98.4% C57BL/6J background. Homozygous DKO (LDLR−/−; ob/ob) mice were generated using classical intercrossing strategies, for which heterozygous ob/+ and C57BL/6 (WT) mice were purchased from Harlan Laboratory. The mice were genotyped by PCR for *LDLR* and *ob* genotype as described before^7^.

The effect of 12-week ACE-I treatment on gene expression in the heart was investigated. In the ACE-I treated groups (DKO-AI and WT-AI) mice were injected with ACE-inhibitor captopril (10 mg/kg/day IP) between 12 and 24 weeks of age. In the control groups (DKO-CO and WT-CO), animals received an equal volume of physiological saline NaCl 0.9%. Hearts were collected at the age of 24 weeks.

### RNA Extraction

Three hearts per group were snap-frozen in liquid nitrogen. Total RNA was extracted using the TRIzol reagent protocol (Invitrogen), purified on an RNeasy spin column (Qiagen), and DNase treated. RNA concentrations were determined using Quant-iT™ RiboGreen® RNA assay kit and RNA purity was assessed on a Nanodrop™ 1000 Spectrophotometer (ThermoFisher Scientific) and RNA integrity using a BioAnalyser 2100 (Agilent Technologies, CA, USA). RIN values were between 9.1 and 10.

### RNA Sequencing

The Genomics Core of UZLeuven-KU Leuven performed gene expression profiling. For all samples 1 µg (50 µl at 20 ng/µl) of total RNA was used as input for the Illumina TruSeq RNA sample prep kit v2 – set A (Illumina, San Diego, USA). Final libraries were quantified using the Qubit High Sensitivity assay (Life Technologies, Carlsbad, USA). Sequencing was done on an Illumina HiSeq. 2000 using the 50 bp single read recipe.

### RNA-Seq Data Analysis

Alignment of sequenced fragments was performed with Tophat^48^ using the *Mus musculus* GRCm38 genome build 10 annotation (Ensembl 75). The read-count per gene was computed with htseq-count and cuffquant, and the differential expression was analyzed with Cufflinks^48^. Cufflinks was used for all further downstream analyses. Genes were considered significantly differentially regulated when fold change was at least 1.5-fold up/down (|log_2_ ratio| > 0.585) between conditions and q-value ≤ 0.05. A Venn diagram of differential gene sets was created with Intervene^49^.

### qRT-PCR and RT-PCR

cDNA was generated using the SensiFAST™ cDNA synthesis kit (Bioline, Meridian Life Science Inc. TN, USA) as per the manufacturer’s protocol.

#### qRT-PCR

Gene expression levels of 20 genes (see Suppl. Table S3 for primer sequences) were determined with beta actin as reference gene. Primers were designed to be exon-exon spanning using Primer-BLAST on NCBI and synthesized by Integrated DNA technologies (Leuven, Belgium). The reaction mixes consisted of 2 μl cDNA, 1 μl primer mix containing forward and reverse primers, 10 μl H_2_O and 10 μl SensiFAST™ SYBR Hi-Rox mix (Bioline, Meridian Life Science Inc. TN, USA). qRT-PCR reactions were run on the Applied Biosystems Step One Plus Real-time PCR system. Transcript levels were normalized using *beta-actin* and mean values statistically compared using T-tests.

#### RT-PCR

Expression of a selection of transcription factors in the heart was validated using RT-PCR and specific primers (see Suppl. Table S6 for primer sequences). Amplification was done using MangoTaq polymerase (Bioline, Meridian Life Science Inc. TN, USA) with PCR cycle parameters: 94 °C 5 min (1x); 94 °C 30 sec, 50 °C 30 sec, 72°C 30 sec (35x); 72°C 7 min (1x) on an Eppendorf Mastercycler ep gradient S. The presence and size of products were verified on a 1% agarose gel and imaged on a VWR^®^ Smart 3 Gel Doc System (VWR International, Leuven, Belgium).

### Pathway and Regulatory Network Analysis

Functional enrichment, pathway, and network analyses were performed using Ingenuity Pathway Analysis (IPA) version 28820210, build 400896 M (Ingenuity Systems Inc., Redwood City, CA). Only molecules from the data set present in the Ingenuity Knowledge Base repository were retained for analysis. Sets of significant differential genes were analyzed by core analysis, resulting in an enrichment analysis of canonical pathways. Enrichment of canonical pathways was scored by Fisher’s exact test. Only pathways of which three or more genes were involved were considered further. For visualization purposes, only parts of canonical pathways where differential gene expression was observed were visualized. Genes found to be involved in canonical pathways were further used to visualize the functional relationship between transcription factors and their effects on pathways through downstream targets. For clarity, large network maps were manually trimmed retaining the nodes with the most connections.

In order to identify upstream regulators based on the observed gene expression changes in our experimental dataset, the IPA Upstream Regulator Analysis was used. To identify candidate regulatory transcription factors involved in regulation of differentially expressed genes, we used the *i-cis* Target^50^ web-based tool that employs large motif collection, does not require an explicit choice of the background regions, relies on scoring based on homotypic clustering of binding sites^51^, uses cross-species information and considers large (10Kb) regulatory space around transcription start sites (TSS) that all together yield high confidence predictions. Lists of up-regulated and of down-regulated genes were used as input. Only TF motifs with Normalized Enrichment Score (NES) above or equal to 2.5 were considered.

### Ethical committee approval

The investigation conformed to the “Guide for the Care and Use of Laboratory Animals” (National Institutes of Health, NIH publication no. 85-23, revised 1996, Bethesda, Md). The Institutional Animal Care Commission and Ethical Committee of the KU Leuven approved all experimental protocols.

## Electronic supplementary material

Supplementary Materials

Supplementary Table S1

## Data Availability

All RNAseq datasets have been uploaded to the NCBI Gene Expression Omnibus. The GEO accession numbers are: GSM3092582-GSM3092593 corresponding to experiment GSE112975 entitled “ACE-inhibition induces a cardioprotective transcriptional response in the metabolic syndrome heart”.
