# Supplementary material for: ACE-inhibition induces a cardioprotective transcriptional response in the metabolic syndrome heart
Source: Sci Rep. 2018 Nov 1;8:16169. doi: 10.1038/s41598-018-34547-9 (PMC6212468; doi:10.1038/s41598-018-34547-9)
Supplement: Supplementary file 1 — Supplementary Materials [file 41598_2018_34547_MOESM1_ESM.pdf]

Supplementary materials

ACE-inhibition induces a cardioprotective transcriptional response in the metabolic syndrome heart.

Aziza Yakubova[1§], Lieven Thorrez[2§], Dmitry Svetlichnyy[3], Liesbeth Zwarts[4], Veerle Vulsteke[4], Griet Laenen[5], Wouter Oosterlinck[1], Yves Moreau[5], Luc Dehaspe[6], Jeroen Van Houdt[6], Álvaro Cortés-Calabuig[6], Bart De Moor[5], Patrick Callaerts[4*], Paul Herijgers[1*]

[1]Department of Cardiovascular Sciences, Research Unit of Cardiac Surgery, KU Leuven, Leuven, Belgium
[2]Department of Development and Regeneration, Interdisciplinary Research Facility, KU Leuven, Campus Kulak Kortrijk, Kortrijk, Belgium
[3]Department of Human Genetics, Laboratory of Computational Biology, KU Leuven, Leuven, Belgium
[4]Department of Human Genetics, Laboratory of Behavioral and Developmental Genetics, KU Leuven, Leuven, Belgium
[5]Department of Electrical Engineering, ESAT - STADIUS, Stadius Centre for Dynamical Systems, Signal Processing and Data Analytics, KU Leuven, Leuven, Belgium
[6]Department of Human Genetics, Genomics Core, Center for Human Genetics, University Hospital, KU Leuven, Leuven, Belgium

[§]joint first authors
*joint corresponding authors

Correspondence to:

Paul Herijgers
email: paul.herijgers@kuleuven.be

Patrick Callaerts
email: patrick.callaerts@kuleuven.be

Supplementary Figure S1 Cardiac hypertrophy signaling

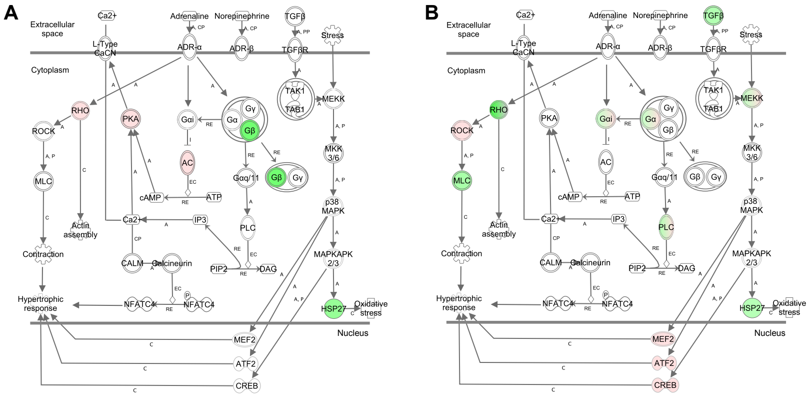

DKO versus WT

ACE-I treatment in DKO

Supplementary Figure S1. Cardiac hypertrophy signaling. (A) Differential gene expression in DKO mice versus WT mice. (B) Differential gene expression in DKO mice after ACE-I. Red=upregulated, green=downregulated. C=causes, E=Expression, EC=Enzyme Catalysis, I=inhibition, L=Molecular cleavage or degradation, O=Oxidized, PP=Protein-Protein binding, RE=Reaction, TR=Translocation.

Supplementary Figure S2 LXR-RXR signaling

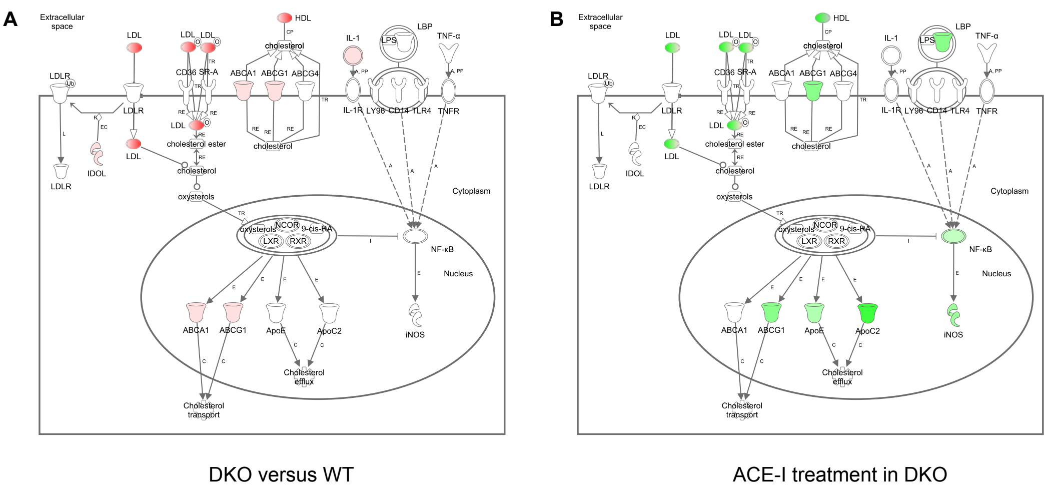

Supplementary Figure S2. LXR-RXR signaling. (A) Differential gene expression in DKO mice versus WT mice. (B) Differential gene expression in DKO mice after ACE-I. The retinoid X receptors (RXRs) are nuclear receptors that mediate the biological effects of retinoids by their involvement in retinoic acid-mediated gene activation. RXRα is the dimerization partner for the type II nuclear receptors that includes the liver X receptor (LXR). The LXR receptor is activated by oxysterol ligands and forms a heterodimer with RXR. After heterodimerization LXR initiates transcription of target genes by binding to the LXR response element. LXR/RXR is involved in the regulation of lipid metabolism, inflammation, and cholesterol to bile acid catabolism. Genes regulated by LXR include ABCA1, ABCG1, ApoE and ApoC2 responsible for cholesterol transport and efflux. LXR/RXR also influences iNOS through NF-kB inhibition. Red=upregulated, green=downregulated. C=causes, E=Expression, EC=Enzyme Catalysis, I=inhibition, L=Molecular cleavage or degradation, O=Oxidized, PP=Protein-Protein binding, RE=Reaction, TR=Translocation.

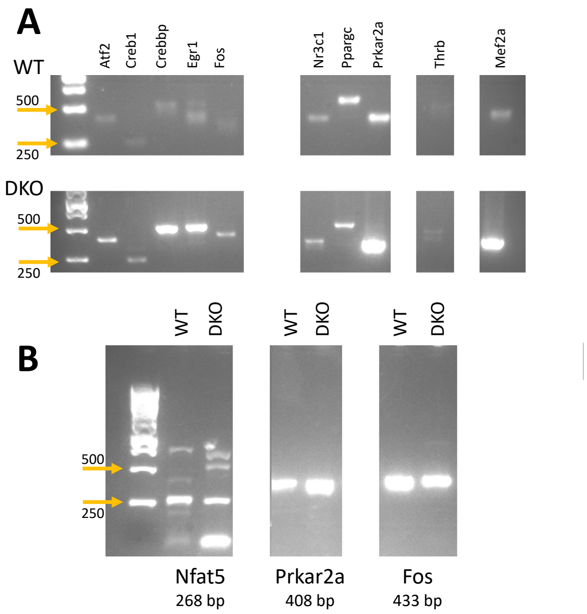

Supplementary Figure S3. RT-PCR results for transcription factor expression in WT and DKO. (A) The observed sizes correspond to the expected size for the various fragments (see Supplementary Table S6). The WT and DKO PCR products were run on two separate gels and the parts of the gels with the relevant band sizes were cropped. The white vertical fields mask lanes on the same gel where no PCR product was detected, this to make the presentation clearer. The original intensity of the gels is displayed. **(B)** Nfat5, Prkar2a and Fos were rerun starting with new cDNA batches to confirm expression. The PCR products for each gene were run on separate gels. The original intensity of the gels is displayed. The arrows mark the 250 bp and 500 bp markers, respectively. Unannotated gels are shown on the following page.

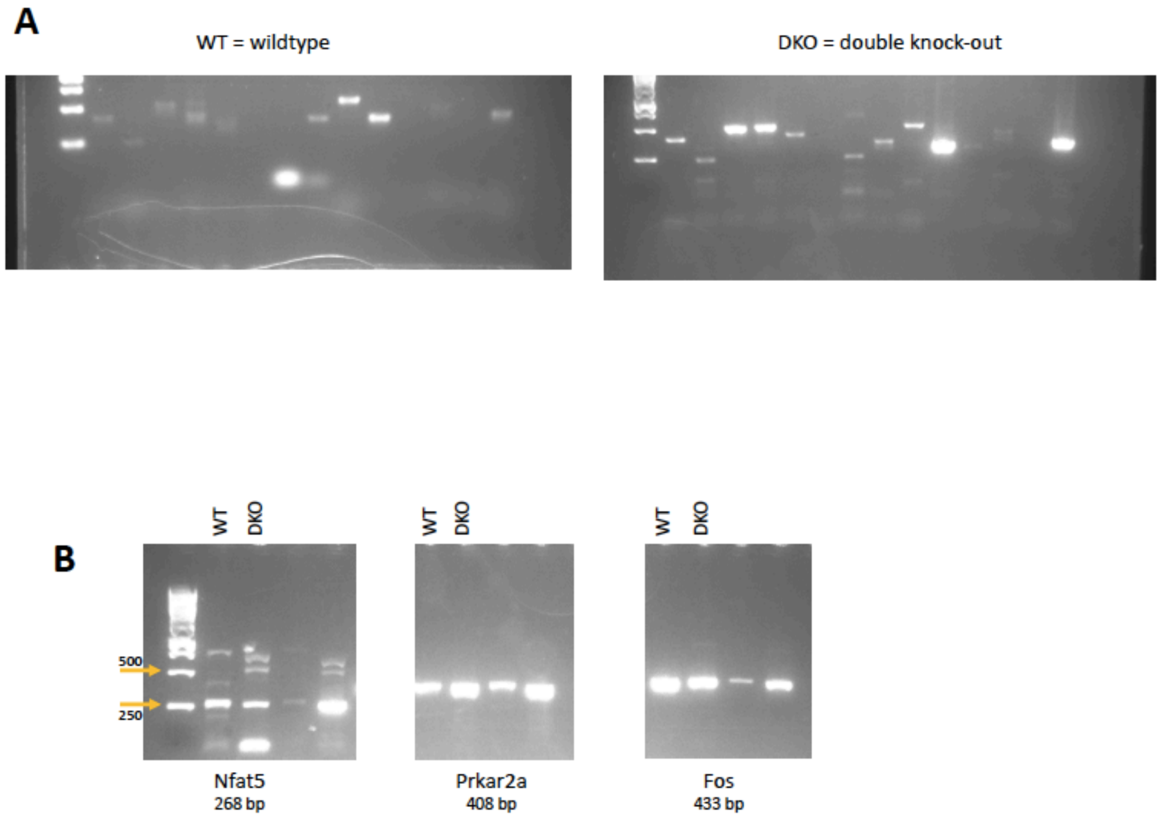

Supplementary Figure S3bis. Unannotated gels corresponding to Suppl. Figure S3.

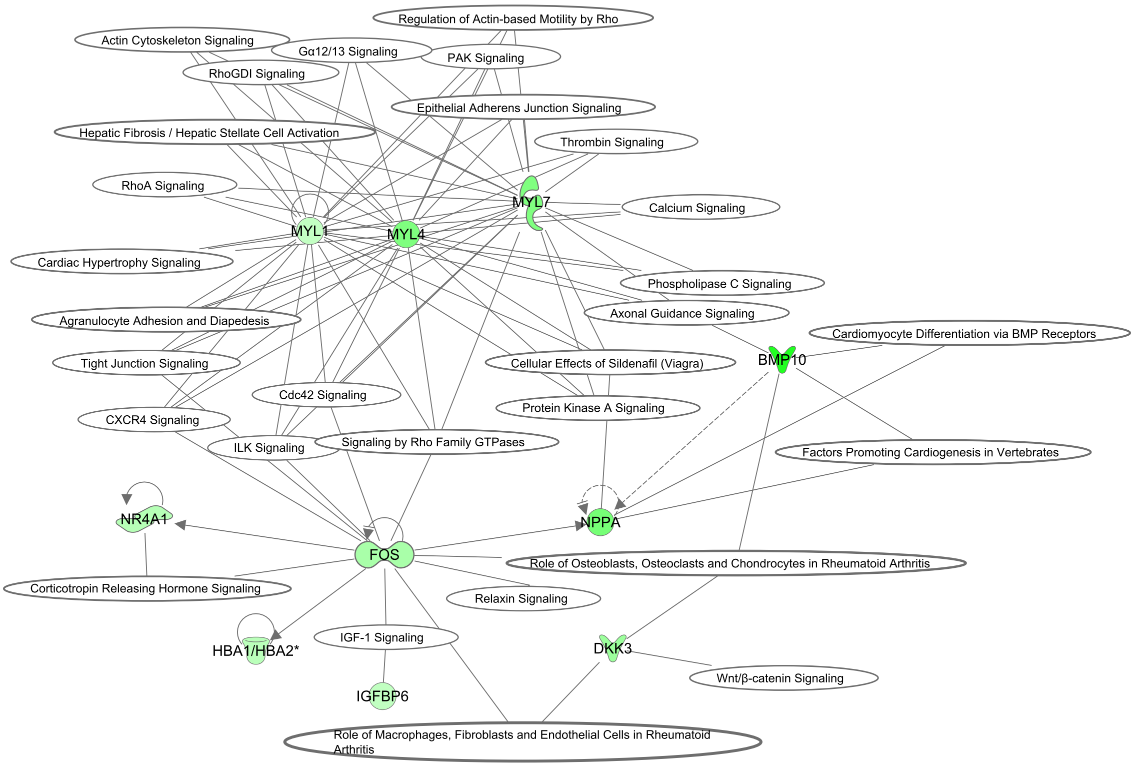

Supplementary Figure S4. Pathways significantly affected by ACE-I in WT. Differential genes are plotted in a network representation to show involvement in pathways. Red and green color indicate up- and downregulation, respectively.

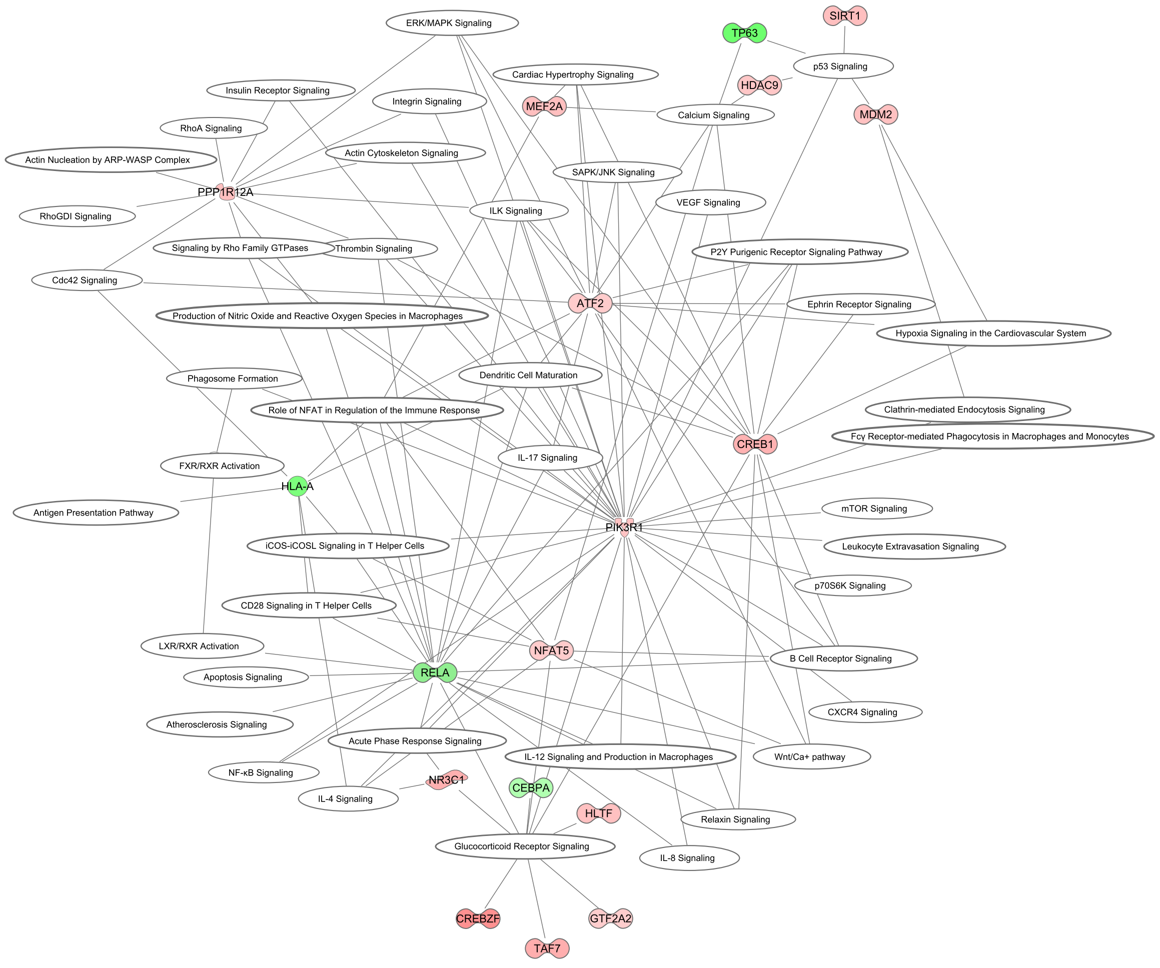

Supplementary Figure S5. Pathways significantly affected by Ace-I treatment in DKO. Differential genes are plotted in a network representation to show involvement in pathways. Red and green color indicate up- and downregulation, respectively.

Supplementary Tables

Supplementary Table S1. 72 significantly enriched pathways identified by Ingenuity Pathway Analysis (IPA).

Supplementary Table S2. Summary table of qRT-PCR analyses of WT versus DKO. Expression levels of genes were normalized to beta-actin and the mean ΔCt values were compared using T-tests. Statistically significant changes are highlighted in bold. In addition, we assigned trends using the following rationale: trend: ▼ if WT/DKO>= 1.5 ▲ if WT/DKO <= 0.5. The fact that a number of values that show clear trends are not statistically significant is probably to the number of biological replicates that were used, i.e. 3. Color coding is the same as in the pathway figures (red= up, green=down).

Gene	WT	DKO	p-value	Trend for DKO
ApoA-1	0.062 ± 0.006	0.0942 ± 0.011	0.234	▲
Abcg1	0.948 ± 0.0132	4.057 ± 0.097	**<0.0001**	▲
PKA	6.620 ± 0.211	11.813 ± 1.491	0.117	=
Fgg	0.026 ± 0.003	1.545 ±0.781	0.324	▲
Fos	7.931 ± 0.669	3.641 ± 0.388	**0.036**	▼
Hsp27	0.855 ± 0.038	1.723 ± 0.259	0.129	=
Hdl	0.246 ± 0.052	0.595 ± 0.090	0.126	=
IL-1	0.273 ± 0.055	2.398 ± 0.330	**0.0215**	▲
RhoA	9.901 ± 0.967	23.42 ± 1.970	**0.0236**	▲
Serpin	6.045 ± 0.191	26.321 ± 3.562	**0.030**	▲
ApoB	0.0374 ± 0.004	0.717 ± 0.366	0.344	▲
Pla2g1b	0.435 ± 0.006	0.852 ± 0.195	0.286	=
Pla2g7	1.069 ± 0.135	3.814 ± 0.195	**0.0025**	▲

Supplementary Table S3. Summary table of qRT-PCR analyses of WT versus WT treated with ACE-I (WT-AI). Expression levels of genes were normalized to beta-actin and the mean ΔCt values were compared using T-tests. Statistically significant changes are highlighted in bold. In addition, we assigned trends using the following rationale: trend: ▼ if WT/DKO>= 1.5 ▲ if WT/DKO <= 0.5. The fact that a number of values that show clear trends are not statistically significant is probably to the number of biological replicates that were used, i.e. 3. Color coding is the same as in the pathway figures (red= up, green=down).

Gene	WT	WT-ACE	P-value	Trend for ACE
Dkk3	1.425 ± 0.380	0.164 ± 0.016	0.128	▼
Fos	9.921 ± 0.873	5.156 ± 1.237	0.143	▼
Lgfbp6	3.403 ± 0.321	3.138 ± 0.194	0.704	=
Myl1	95.157± 15.90	54.74 ± 6.91	0.249	▼
Myl4	0.196 ± 0.026	0.116 ± 0.025	0.270	▼
Myl7	343.4 ± 40.8	116.1 ± 40.7	0.085	▼
Nr4a1	24.169 ± 7.541	14.358 ± 2.355	0.513	▼

Supplementary Table S4. Summary table of qRT-PCR analyses of DKO versus DKO treated with ACE-I (DKO-AI). Expression levels of genes were normalized to beta-actin and the mean ΔCt values were compared using T-tests. Statistically significant changes are highlighted in bold. In addition, we assigned trends using the following rationale: trend: ▼ if WT/DKO>= 1.5 ▲ if WT/DKO <= 0.5. The fact that a number of values that show clear trends are not statistically significant is probably to the number of biological replicates that were used, i.e. 3. Color coding is the same as in the pathway figures (red= up, green=down).

Gene	DKO	DKO-ACE	p-value	Trend for ACE
ApoA-1	0.074 ± 0.009	0.249 ± 0.065	0.200	▲
Abcg1	2.634 ± 0.063	3.028 ± 0.411	0.615	=
PKA	7.670 ± 0.968	14.87 ± 1.900	0.123	=
Dkk3	2.375 ± 0.734	0.319 ± 0.100	0.184	▼
Fgg	1.003 ± 0.507	0.046 ± 0.008	0.337	▼
Fos	2.365 ± 0.252	2.186 ± 0.411	0.841	=
Hsp27	1.119 ± 0.168	1.740 ± 0.275	0.329	=
Hdl	0.423 ± 0.064	0.955± 0.162	0.154	=
Igfbp6	3.519 ± 0.085	6.310 ± 1.878	0.439	=
Il-1	1.737 ± 0.239	0.056 ± 0.013	**0.015**	▼
Myl1	140.7 ± 34.04	116.4 ± 23.86	0.752	=
Myl4	0.313 ± 0.071	0.503 ± 0.123	0.483	=
Myl7	391.2 ± 100.1	585.6 ± 153.9	0.574	=
Myl9	30.46 ± 5.023	28.68 ± 5.188	0.893	=
RhoA	16.71 ± 1.405	21.92 ± 4.208	0.534	=
Rictor	2.276 ± 0.467	5.293 ± 0.820	0.139	▲
Serpin	18.78 ± 2.541	18.09 ± 2.579	0.918	=
ApoB	0.512 ± 0.261	0.102 ± 0.020	0.418	▼
Pla2g1b	0.608 ± 0.139	0.360 ± 0.019	0.368	▼
Pla2g7	2.721 ± 0.139	2.353 ± 0.532	0.719	=

Supplementary Table S5. Summary table of qRT-PCR primers

Gene	Forward primer	Reverse primer
ApoA-1	TGTGGATGCGGTCAAAGACA	TCCAGGAGATTCAGGTTCAGC
Abcg1	AAGGTCTCCAATCTCGTGCC	TCCATGACAAAGTCTGCTGGG
Pka	CGAGCAGGAGAGCGTGAA	CCTTGTGCTTCACCAGCATC
Dkk3	GGCAAGAGGAGCCATGAATGT	CCACCTTTGGTGGCCTTTTG
Fgg	GACGGCATTATTTGGGCGAC	AACGTCTCCAGCCTGTTTGG
Fos	TTTCAACGCCGACTACGAGG	TCTGCGCAAAAGTCCTGTGT
Hsp27	GGCAGTACTTGGGATCAGGG	CAGGGGTCCACATCTTCTCC
Hdl	CCTTCCTGTGTGGTCGTGA	TCAGCCTGCCAGCTTTTACTTC
Igfbp6	CCGTCGGAGGAGACTACAAAG	GTGGATTCTTCTGCCGGTCT
IL-1	GCTTGAGTCGGCAAAGAAATCA	AGATGGTCAATGGCAGAACTGT
Myl1	CGGAGTTTTCAAGCACGCAA	TCTGCATGGTGGTAAGCTGG
Myl4	TATCAACGGCTCAAGTCGGC	GGTGGGACCTCACTCATCTCA
Myl7	GGCACAACGTGGCTCTTCTA	ACACTTACCCTCCCGAGCTG
Myl9	CTCTGCAGCAGGGAAACC	CATGGCGAAGACATTGGACG
Nr4a	ATGGAGCTGGAACAAACGCC	TTGAATACAGGGCATCTCTGGG
RhoA	GGTCCTCCGTCGGTTCTCTC	GATGCAAGGCTCAAGGCGAG
Rictor	AATGCACCCGTCCTTGTCTC	TCATAAACCTGCTTGGCGTC
Serpin	ACAACCCGACAGAGACAATCC	TTCGTCCCAAATGAAGGCGT
ApoB	TACTTCCACCCACAGTCCCCT	CCTTAGAAGCCTTGGGCACAT
Pla2g1b	GGACGACTTAGACAGGTGCT	GTGTTGGTGTAGGGGTTGTCT
Pla2g7	GAGGCTGTATGCTCAACCCA	GGCTTCAGTTTGATGTTCTGGT

Supplementary Table S6. Summary table of RT-PCR primers

Gene	Forward primer	Reverse primer	Expected size
Atf2	GTGGCCAGCGTTTTACCAAC	GCTGGACGAACGATAGCTGA	406
Creb1	GAGGTGTAGTTTGACGCGGT	TGAGCTGCTGGCATGGATAC	243
Crebbp	GCCCATTGTGCATCTTCACG	GCTGCTGTAGGTATCGTGCT	524
Egr1	TTACCCGCCATATCCGCATC	CTGGGAGAAAAGGTCGCTGT	526
Fos	TGCCAACTTTATCCCCACGG	TCGGTGGGCTGCCAAAATAA	433
Foxo3	GAGCTGGAGCTCGAACCTT	GGAGCATTTCCTTGGTTGCC	561
Nfat5	CCTCTGAAGCAGGGAGTGTC	CTTCGGGGTTGATGGATGCT	268
Nr3c1	GTGGAAGGACAGCACAATTACC	ATCCTGGTATCGCCTTTGCC	403
Ppargc1	ACTCTCAGTAAGGGGCTGGT	TAGCTGAGCTGAGTGTTGGC	563
Prkar2a	CTCCAGCACTGTAACAGCCA	GCCCAATGCACCTCTTACCT	408
Thrb	CGCTCTGATCCGTGTTTTCC	CGATAGTGGTACCCTGTGGC	490
Rela	GCTGCGGAGCTTGTAGTCG	TGCTTCGGCTGTTCGATGAT	392
